# Complete Genome Sequence of the Uropathogenic Methicillin-Resistant Staphylococcus aureus Strain MRSA-1369

**DOI:** 10.1128/mra.00981-22

**Published:** 2022-09-29

**Authors:** Hasan Tükenmez, Taylor M. Nye, Mari Bonde, Michael G. Caparon, Fredrik Almqvist, Scott J. Hultgren, Jörgen Johansson

**Affiliations:** a Department of Chemistry, Umeå University, Umeå, Sweden; b Umeå Centre of Microbial Research, Umeå University, Umeå, Sweden; c Molecular Infection Medicine, Sweden, Umeå University, Umeå, Sweden; d Department of Molecular Biology, Umeå University, Umeå, Sweden; e Department of Molecular Microbiology, Washington University School of Medicine, St. Louis, Missouri, USA; f Center for Women's Infectious Disease Research, Washington University School of Medicine, St. Louis, Missouri, USA; g QureTech Bio, Umeå, Sweden; University of Rochester School of Medicine and Dentistry

## Abstract

MRSA-1369 is a uropathogenic methicillin-resistant Staphylococcus aureus (MRSA) strain. Here, we present the complete genome sequence of MRSA-1369, which consists of one chromosome (2.87 Mb) and two plasmids (16.68 kb and 3.13 kb). This will serve as a reference genome for future Staphylococcus aureus pathogenesis and multiomic studies.

## ANNOUNCEMENT

Methicillin-resistant Staphylococcus aureus (MRSA) is one of the major causes of health care-associated infections, including skin and soft tissue infections, pneumonia, bacteremia, endocarditis, urinary tract infections (UTIs), and catheter-associated UTIs (CAUTIs) (1, 2). MRSA-1369 was isolated from a urine sample from a CAUTI patient in Barnes-Jewish Hospital (St. Louis, MO, USA) (exempt from ethics committee review) and was confirmed as S. aureus by matrix-assisted laser desorption ionization–time of flight mass spectrometry (MALDI-TOF MS). The mechanism of urinary catheter biofilm formation and the progression to symptomatic CAUTI have been studied using this strain (3). Here, we report the complete genome sequence of the MRSA-1369 strain, which was assembled using short reads from Illumina sequencing (30× coverage) and long reads from Oxford Nanopore Technologies (ONT) sequencing (27× coverage) for total genome coverage of 57×.

MRSA-1369 was grown on brain heart infusion (BHI) agar (BD Biosciences) overnight at 37°C. A lawn of colonies was collected, washed with 1× phosphate-buffered saline (PBS), and then suspended in 500 μL of 1× DNA/RNA shield buffer (Zymo Research, USA). DNA was prepared by MicrobesNG (Birmingham, UK) as described previously (4) and used for both Illumina and Nanopore library preparations.

**FIG 1:**
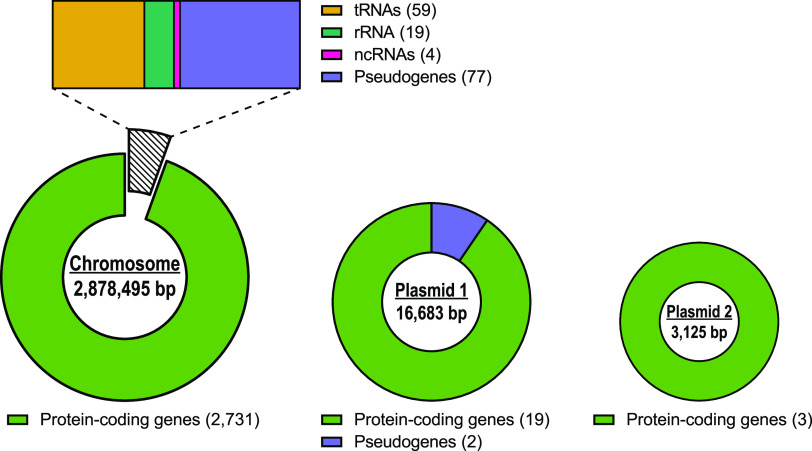
Main features of the MRSA-1369 genome annotation. Genome annotations were determined by NCBI PGAP (7). The number of features in each category is indicated in parentheses.

Illumina sequencing libraries were prepared using the Nextera XT library preparation kit (Illumina, USA) according to the manufacturer’s protocol except that input DNA was increased 2-fold and the elongation time was increased to 45 s. Libraries were sequenced with the Illumina NovaSeq 6000 platform using the 250-bp paired-end protocol. Trimmomatic v0.30 was used to trim adapters from the Illumina reads with a sliding window quality cutoff score of Q15 (5), producing 542,755 paired-end reads of up to 300 bp. Long-read libraries were prepared with the SQK-RBK004 kit (ONT, UK) using 400 to 500 ng of high-molecular-weight DNA without shearing or size selection and were loaded in a FLO-MIN106 (R.9.4.1) flow cell in a GridION system (ONT). Adapter trimming was performed with Guppy v5.1.13 during the base-calling process. Read lengths were 22,265.9 bp (mean) and 8,794.0 bp (median), with an average read quality value of 13.1 over 3,659 total reads and a read *N*_50_ value of 54,581.0 bp. Illumina and Nanopore reads were assembled using Unicycler v0.4.0 (6), which yielded a final assembly of 3 circular contigs with genome coverage of 57.43×. Contigs were annotated using the NCBI Prokaryotic Genome Annotation Pipeline (PGAP) v6.1 (7). Default software parameters were used unless otherwise specified. The complete MRSA-1369 genome sequence consists of one large circular chromosome of 2,878,496 bp (GC content, 33%), rotated to start at *dnaA*, and two plasmids of 16,683 bp (GC content, 29%) and 3,125 bp (GC content, 29%). According to the NCBI PGAP annotation, the MRSA-1369 genome, including both plasmids, contains 2,914 genes, with 2,753 protein-encoding genes, 59 tRNAs, 19 rRNAs, and 4 noncoding RNAs (Fig. 1).

Using the Comprehensive Antibiotic Resistance Database (CARD) and the Resistance Gene Identifier (RGI), we identified several putative MRSA-1369 genes with high sequence identity (>95%) to known antimicrobial resistance (AMR) genes (8) (Table 1). The MRSA-1369 genome will enable further investigation of multidrug resistance mechanism(s), future multiomic experiments, and studies of CAUTI pathogenesis in the murine model.

**TABLE 1:** Determination of AMR genes in the MRSA-1369 genome sequence

Replicon	Nucleotide start position	Nucleotide stop position	Hit gene^*a*^	Hit identity (%)^*a*^	AMR gene family^*a*^
Chromosome	38584	40590	*mecA*	99.7	Methicillin-resistant PBP2
Chromosome	2457648	2458067	*fosB*	100	Fosfomycin thiol transferase
Plasmid 1	11549	12394	*blaZ*	96.8	BlaZ β-lactamase
Chromosome	2223805	2225070	*murA* ^*b*^	99.3	Antibiotic-resistant MurA transferase
Chromosome	117343	118731	*norC*	98.9	MFS antibiotic efflux pump
Chromosome	387148	387567	*mepR*	100	MATE transporter
Chromosome	387674	389029	*mepA*	100	MATE transporter
Chromosome	765292	765735	*mgrA*	100	ABC antibiotic efflux pump; MFS antibiotic efflux pump
Chromosome	773386	774552	*norA*	99.7	MFS antibiotic efflux pump
Chromosome	1459272	1460627	*arlS*	100	MFS antibiotic efflux pump
Chromosome	1460624	1461283	*arlR*	100	MFS antibiotic efflux pump
Chromosome	2304458	2305903	*lmrS*	99.8	MFS antibiotic efflux pump
Chromosome	2306222	2306695	*sepA*	96.8	SMR antibiotic efflux pump
Chromosome	2306794	2308137	*sdrM*	100	MFS antibiotic efflux pump

a AMR gene hits were determined using the CARD and RGI tools. PBP2, penicillin-binding protein 2; MFS, major facilitator superfamily; MATE, multidrug and toxic compound extrusion; ABC, ATP-binding cassette; SMR, small multidrug resistance.

b Encodes type II MurA with a G257D amino acid substitution.

### Data availability.

The complete genome sequence of the MRSA-1369 strain has been deposited in GenBank under the accession numbers CP099576 to CP099578. The raw sequence reads have been deposited in the SRA under the accession numbers SRR19786624 (ONT) and SRR19786625 (Illumina). The associated BioProject and BioSample accession numbers are PRJNA851804 and SAMN29251701, respectively.
